# The genetics of smoking in individuals with chronic obstructive pulmonary disease

**DOI:** 10.1186/s12931-018-0762-7

**Published:** 2018-04-10

**Authors:** Ma’en Obeidat, Guohai Zhou, Xuan Li, Nadia N. Hansel, Nicholas Rafaels, Rasika Mathias, Ingo Ruczinski, Terri H. Beaty, Kathleen C. Barnes, Peter D. Paré, Don D. Sin

**Affiliations:** 1 The University of British Columbia Center for Heart Lung Innovation, St Paul’s Hospital, Vancouver, BC Canada; 2 Pulmonary and Critical Care Medicine, School of Medicine, Johns Hopkins University, Baltimore, MD USA; 3 Division of Biomedical Informatics and Personalized Medicine, Department of Medicine, University of Colorado School of Medicine, Anschutz Medical Campus, Aurora, CO USA; 4 Division of Genetic Epidemiology, School of Medicine, Johns Hopkins University, Baltimore, MD USA; 5 Department of Biostatistics, Bloomberg School of Public Health, Johns Hopkins University, Baltimore, MD USA; 6 Department of Epidemiology, Bloomberg School of Public Health, Johns Hopkins University, Baltimore, MD USA; 7 Respiratory Division, Department of Medicine, University of British Columbia, Vancouver, BC Canada

**Keywords:** Cessation, Smoking, GWAS, eCO, Cotinine

## Abstract

**Background:**

Smoking is the principal modifiable environmental risk factor for chronic obstructive pulmonary disease (COPD) which affects 300 million people and is the 3rd leading cause of death worldwide. Most of the genetic studies of smoking have relied on self-reported smoking status which is vulnerable to reporting and recall bias. Using data from the Lung Health Study (LHS), we sought to identify genetic variants associated with quantitative smoking and cessation in individuals with mild to moderate COPD.

**Methods:**

The LHS is a longitudinal multicenter study of mild-to-moderate COPD subjects who were all smokers at recruitment. We performed genome-wide association studies (GWASs) for salivary cotinine (*n* = 4024), exhaled carbon monoxide (eCO) (*n* = 2854), cigarettes per day (CPD) (*n* = 2706) and smoking cessation at year 5 follow-up (*n* = 717 quitters and 2175 smokers). The GWAS analyses were adjusted for age, gender, and genetic principal components.

**Results:**

For cotinine levels, SNPs near *UGT2B10* gene achieved genome-wide significance (i.e. *P* < 5 × 10^− 8^) with top SNP rs10023464, *P* = 1.27 × 10^− 11^. For eCO levels, one significant SNP was identified which mapped to the *CHRNA3* gene (rs12914385, *P* = 2.38 × 10^− 8^). A borderline region mapping to *KCNMA1* gene was associated with smoking cessation (rs207675, *P* = 5.95 × 10^− 8^). Of the identified loci, only the CHRNA3/5 locus showed significant associations with lung function but only in heavy smokers. No regions met genome-wide significance for CPD.

**Conclusion:**

The study demonstrates that using objective measures of smoking such as eCO and/or salivary cotinine can more precisely capture the genetic contribution to multiple aspects of smoking behaviour. The *KCNMA1* gene association with smoking cessation may represent a potential therapeutic target and warrants further studies.

**Trial registration:**

The Lung Health Study ClinicalTrials.gov Identifier: NCT00000568. Date of registration: October 28, 1999.

**Electronic supplementary material:**

The online version of this article (10.1186/s12931-018-0762-7) contains supplementary material, which is available to authorized users.

## Background

The smoking epidemic is one of the biggest public health challenges in modern history [1]. Tobacco-attributable deaths are expected to rise to more than 10 million globally by 2030 [2–4]. Despite aggressive public health programs aimed at eliminating smoking in the United States (US) and elsewhere, one in 4 adults in the US still use tobacco products and 1 in 5 are daily users. Smoking is the principal modifiable environmental risk factor for chronic obstructive pulmonary disease (COPD), ischemic heart disease, and lung cancer [5]. COPD, for instance, affects 300 million people and is the 3rd leading cause of death worldwide [6].

Genetic studies of smoking behaviour and smoking-related illnesses such as COPD and lung cancer have identified strong associations in the chromosome 15q25 region, which contains genes encoding the nicotinic receptor subunits *CHRNA3-CHRNA5-CHRNB4* [7–9]. However, most of these studies have relied on self-reported smoking status as either the phenotype of interest or one of the covariates to “adjust” tobacco exposure. Self-report is vulnerable to reporting and recall bias and has been shown to consistently underestimate total tobacco exposure [10, 11], which may result in residual confounding [12]. One way to mitigate this risk is to use an objective biochemical assay to validate self-reports of tobacco use. The most commonly used assays include exhaled carbon monoxide (eCO) [13] or cotinine in serum, urine or saliva [14]. Because eCO has a short half-life (~ 4 h) it is best suited for short term tobacco exposure while the longer half-life of cotinine (~ 16 h) makes it a more robust measure to differentiate active from non-active smokers with a longer duration of abstinence. However, because cotinine is a by-product of nicotine, enzymatic processes involved in nicotine metabolism can affect cotinine levels in smokers. eCO and/or cotinine assays are relatively inexpensive, non-invasive and well-standardized measurements and most importantly they more accurately quantify tobacco exposure in smokers compared with self-report alone [13, 15].

To date, very few studies have ascertained the genetic drivers of smoking (and cessation) using validated biochemical assays [16], especially among those with established smoking related diseases such as COPD. Using data from the Lung Health Study, we identified the genetic variants associated with cigarette smoking using validated objective assays in individuals with mild to moderate COPD.

## Methods

### The lung health study (LHS)

The details of the LHS have been published previously [17, 18]. Briefly, LHS was a multicenter clinical study that evaluated the effects of ipratropium bromide, a short acting antimuscarinic agent (i.e. ipratropium bromide), and smoking cessation on lung function decline in current smokers with mild to moderate COPD. At the time of recruitment all subjects were active smokers between the ages of 35 and 60 years (with a mean age of 48 years) who had smoked at least 10 cigarettes a day within the 30 days prior to initial screening and who demonstrated mild to moderate COPD on spirometry defined by forced expiratory volume in 1 second (FEV_1_) between 55% and 90% of predicted, in the presence of a FEV_1_/forced vital capacity (FVC) ratio of < 0.70 after bronchodilation. The mean FEV_1_ of the cohort was 75% predicted and the mean FEV_1_/FVC was 63% post-bronchodilator.

After enrolment, these patients were randomly assigned to one of 3 groups: (1) usual care (UC), who received no intervention, *n* = 1964; (2) an intense anti-smoking (special) intervention and ipratropium bromide (Atrovent®, Boehringer Ingelheim Pharmaceuticals) *n* = 1961 (SIA); or (3) an intense anti-smoking (special) intervention and an inhaled placebo, *n* = 1962 (SIP). Ten centers participated in the original study and together they recruited 5887 patients (of whom 37% were females). Those who were in the SIP or SIA groups received a program that consisted of: 1) a strong recommendation by attending physician for smoking cessation in an one-on-one encounter; 2) a group program led by a health educator that met 12 times over 10 weeks, which taught behavioural modification techniques; and 3) nicotine replacement therapy with nicotine gum (2 mg per piece, Nicorette Gum, Marion Merrell Dow Inc), which was provided at no cost to patients. Those who successfully quit smoking were enrolled in a maintenance program to prevent relapses.

For the first 5 years, the lung function of participants was measured annually. At each face to face visit the subjects’ smoking status was determined using a questionnaire, which was validated by salivary cotinine and exhaled carbon monoxide levels as previously described [18]. Participants were classified as smokers if their cotinine levels were greater than 20 ng/mL or if their exhaled carbon monoxide concentrations were higher than 10 ppm. At year 5 of the study, participants were divided into three groups based on smoking history as previously described [19]. Sustained quitters (SQs) were defined as those who gave a history of total abstinence (no month in which the subject smoked even a single cigarette per day) and had eCO readings below 10 ppm at each annual follow-up visit over 5 years. Continuing smokers (CSs) were those who reported smoking at all scheduled follow-up visits. Intermittent quitters (IQs) were current smokers at some but not all of their visits. Given the ambiguity of the IQ group (*n* = 1210) in terms of cigarette smoking, they were excluded from the smoking cessation genome wide association study (GWAS).

#### Genotyping

At year 5 of LHS, venipuncture was carried out on 5413 LHS participants who were alive and eligible at this visit. Blood samples were taken when participants were stable and free of exacerbations for at least 4 weeks and were separated into buffy coat and serum [20]. DNA was extracted from the buffy coat samples of 4251 European Americans in LHS and SNP genotyping was performed. The details of genotyping and quality control have been previously described [21]. Briefly, samples were genotyped using the Illumina Human660WQuad v.1_A BeadChip. Overall, 98.4% of samples (*n* = 4181) passed initial quality control standards and genotypes were available for 559,766 SNPs. An additional 133 samples were removed because they failed subsequent quality control, which resulted in a final sample of *n* = 4048 for the present analysis. Imputation was undertaken with the Michigan Imputation Server [22] using the Haplotype Reference Consortium (HRC) [23] panel. Variants were excluded if the imputation r^2^ was < 0.5 and if the minor allele frequency was < 1%.

### Measurements of expired carbon monoxide (eCO) and cotinine

The details of eCO and cotinine measures have been previously described [24]. To conduct the cotinine assay, LHS participants were asked to deposit at least 1 ml of saliva in a plastic vial, which was then frozen and sent in a batch to the American Health Foundation laboratory in Valhalla, NY. One sample was taken from each participant and a single cotinine assay measurement was performed. The cotinine assessment was conducted using the radioimmunoassay technique of Langone et al. by personnel who were blinded to the smoking status of the participants [24]. In LHS, the sensitivity and specificity of using a salivary cotinine cutoff of 20 ng/ml compared to self-report was 99% and 92%, respectively. Studies have reported a technical coefficient of variation (CV) value of 5% for salivary cotinine assay [25].

Carbon monoxide in expired air was measured using either of two instruments: the MiniCO (Catalyst Research) or the EC50 (Vitalograph). The eCO measurement procedure involved two attempts. If the two values were not within 4 ppm, the measurements were repeated. The result was the average of the two readings rounded to the nearest integer [24].

### Genome-wide association analyses (GWASs)

Given that eCO, cotinine and cigarettes per day (CPD) distributions were skewed, the values were transformed into normally distributed Z scores using the R function ‘rntransform’ [26] (Additional file 1: Figure S1). We performed GWAS for three transformed phenotypes: eCO, salivary cotinine and CPD using SNPTEST [27] assuming an additive genetic model and adjusting for age, gender, and the first 5 genetic principal components (PCs). Since cotinine levels were measured in all subjects at baseline visit, we performed the GWAS for cotinine baseline levels to make available the largest sample size (*n* = 4024 and missing data rate = 1.9%). The eCO and CPD values were only measured in smokers at subsequent visits so we used the values measured at year 1 follow up to make available the largest sample size (*n* = 2706 and missing data rate = 8.1% for eCO; *n* = 2854 and missing data rate = 3.12% for CPD). Significant SNPs were defined as the sentinel SNPs meeting genome-wide significance (*P* < 5 × 10^− 8^).

### Evaluation of previously published variants which relate to smoking cessation

We evaluated previously published hits from two studies for smoking cessation. The first study is the Tobacco and Genetic Consortium (TAG) meta-analysis GWAS of smoking behaviour [28]. The TAG study included 41,278 former and current smokers and the top 15 associated SNPs for self-reported smoking cessation were followed up in 64,924 independent individuals from the European Network of Genetic and Genomic Epidemiology (ENGAGE) and the Oxford-GlaxoSmithKline (Ox-GSK) consortia. This meta-analysis identified only one significant SNP as being associated with smoking cessation; rs3025343 (*P* = 1.8 × 10^− 8^), which was located on chromosome 9, near the dopamine beta-hydroxylase (DBH) gene.

The second study by Siedlinski et al. reported GWAS results for self reported phenotypes of smoking including lifetime average and current CPD, age at smoking initiation, and smoking cessation in 3441 patients with COPD [29]. In total, the meta-analysis included 1164 current smokers and 1907 former smokers (all using self report of yes/no answers); none of the SNPs had showed a statistically significant association with smoking cessation. In the present study, we interrogated the 9 SNPs associated with smoking cessation reported in Siedlinski et al. study.

### Association of smoking related SNPs with extremes of lung function

To determine whether any of the smoking related SNPs discovered in the present study also had an impact on smoking-related physiological outcomes such as lung function, we tested SNPs identified in this study for association with lung function in never and, separately, in heavy smokers. The UK Biobank Lung Exome Variant Evaluation (UK BiLEVE) evaluated the genetic determinants related to low (mean of 65.6% predicted, average (mean of 90.6% predicted), or high (mean of 118% predicted) forced expiratory volume in 1 second (FEV_1_) in heavy smokers (mean 35 pack-years) and separately in never smokers. The study included 10,002 individuals with low FEV_1_, 10,000 with average FEV_1_, and 5002 with high FEV_1_ from each of the heavy smoker and never smoker groups. Genome-wide genotyping was performed using a custom Affymetrix Axiom array (UK BiLEVE array; Santa Clara, CA, USA). After quality control non-genotyped variants were imputed using a combined 1000G Phase 1 and UK10K Project [30] reference panel.

### Gene drug interactions

To uncover the potential biological relevance of the smoking cessation GWAS hits, we used two databases to search for potential gene drug interactions: the DGIdb [31] http://www.dgidb.org/ and the DRUGBANK database [32] https://www.drugbank.ca/ .

## Results

### Descriptive demographics of LHS participants

**Fig. 1:**
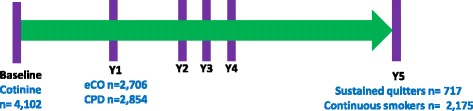
Overall LHS smoking GWAS study design. eCO: exhaled carbon monoxide. CPD: Cigarettes per day. Y1: year 1. eCO was measured in those reporting current smoking

The overall LHS design and the sample size for each of the GWASs are shown in Fig. 1, and the demographics and quantitative smoking values are shown in Table 1. Among smokers at year 1 of LHS, there was a strong correlation between eCO, cotinine, and CPD values with the correlation between eCO and CPD being the strongest (*r* = 0.5, *P* = 1 × 10^− 170^, Additional file 1: Figure S2).

**Table 1:** Demographics of study subjects. Gender male in n (% of column totals), other variables in mean±SD. CPD: cigarettes per day. eCO exhaled carbon monoxide. * Age at assessment

	Baseline	Year 1		Year 5		
	Smokers	Smokers	Quitters	Continuous smokers	Sustained quitters	Intermittent quitters
N (%)	4102	2946	1156	2175	717	1210
Age* (years)	48.6 ± 6.7	49.4 ± 7	50.0 ± 6.7	53.3 ± 6.7	54.2 ± 6.6	53.7 ± 6.7
Gender (male)	2853 (63%)	1837 (62%)	746 (65%)	1366 (63%)	481 (67%)	736 (61%)
BMI (kg/m^2^)	25.5 ± 3.8	25.7 ± 3.9	27.3 ± 4.0	26.0 ± 4.2	28.4 ± 4.3	27.5 ± 4.2
FEV1% predicted	78.6 ± 9.0	78.0 ± 9.8	81.8 ± 9.5	72.8 ± 12.0	80.3 ± 10.9	77.2 ± 11.5
eCO (ppm)	32.4 ± 16.1	24.8 ± 13.2	4.8 ± 2.5	25.4 ± 13.8	4.0 ± 2.4	9.6 ± 11.1
Cotinine (ng/ml)	361.4 ± 199.3	302.7 ± 146.3	92.2 ± 158.7	343.1 ± 195.3	27.8 ± 132.4	117.5 ± 229.6
CPD cigarettes/day	21.9 ± 14.5	21.9 ± 14.5	0 ± 0	23.0 ± 12.9	0 ± 0	5.3 ± 10.2

The levels of quantitative smoking biomarkers such as cotinine were inversely related to cross sectional lung function measures at years 1–5 (Additional file 1: Figure S3). This relationship was significant (*P* < 0.05) for years 1–4 and borderline significant in year 5 (*P* = 0.08). Furthermore, cotinine levels at year 5 showed negative correlation with FEV1 decline between years 1 and 5 (*P* = 3 × 10^− 11^, Additional file 1: Figure S4).

### Genome-wide association results for cotinine levels

In the four GWAS analyses, we included 7,807,992 variants with MAF > 1% and imputation quality> 0.5. The GWAS of cotinine levels at baseline included 4024 individuals. Quantile–quantile (QQ) plots are presented in Additional file 1: Figure S5, which showed a sharp deviation from the expected distribution for low *p*-values indicating strong signals. The genomic inflation factor (λ) was 0.996, suggesting no systematic deviation in the association statistics due to factors such as population structure.

**Fig. 2:**
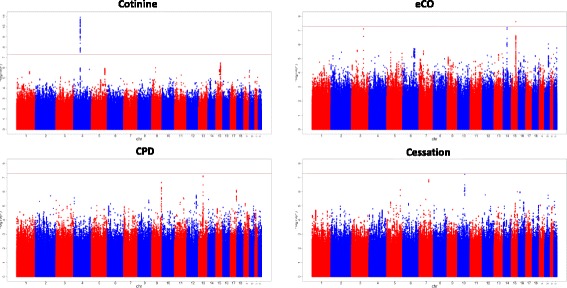
Manhattan plots of smoking GWASs in Lung Health Study. The plots show the *P* values (−log10 scale) on the Y axes and the SNP positions across 22 autosomal chromosomes on the X axes. The horizontal red line represents the genome-wide cut-off of 5 × 10^− 08^

**Fig. 3:**
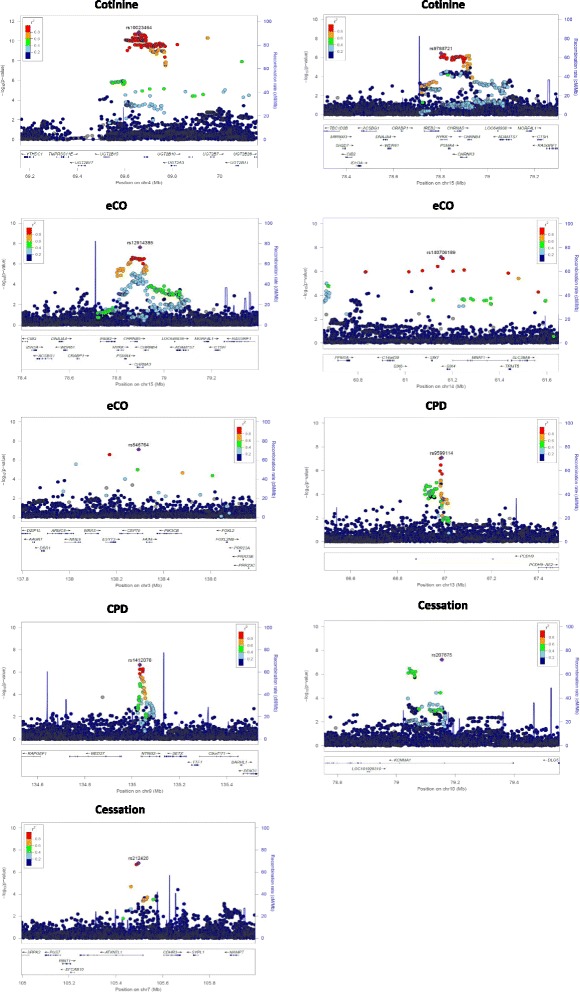
Region plots of the smoking associated loci. The Y axis represent the *P* values in the (−log10 scale) and the X axis is the genomic position. Gene names and their corresponding coordinates are shown below. The sentinel SNP is shown as a purple diamond and the color coding of SNPs reflects the degree of linkage disequilibrium (LD) with the sentinel SNP using 1000G reference

A total of 250 SNPs in the 4q13.2 region containing the UGT2B10 gene achieved genome-wide significance (*P* < 5 × 10^− 8^). A Manhattan plot is shown in Fig. 2 and the region plots are shown in Fig. 3. The most significantly associated SNP for cotinine levels was an intergenic SNP on chromosome 4 (rs10023464) (*P* = 1.27 × 10^-11^). Another interesting signal was observed on the 15q25.1 region with an intronic SNP rs9788721 (*P* = 3.49 × 10^-7^) in the HYKK gene near CHRNA3/5 genes, though this latter association did not reach genome-wide significance. All SNPs meeting or approaching genome-wide significance for smoking phenotypes are presented in Table 2.

**Table 2:** Genetic loci associated with smoking behaviour in the Lung Health Study

Phenotype	SNP	Chr	Gene(s)	Position (hg19)	Alleles (REF/ALT)	MAF	Imputation r2	beta	SE	P value	% Variance
Cotinine	rs10023464	4	UGT2B10	69,659,738	C/T	9.8%	0.995	0.25	0.04	1.27e-11	1.1%
Cotinine	rs9788721	15	CHRNA3/5	78,802,869	C/T	39.5%	0.989	−0.12	0.02	3.49e-7	0.6%
eCO	rs546764	3	CEP70	138,294,336	T/G	2.8%	0.844	0.39	0.07	7.76e-8	0.9%
eCO	rs140706189	14	SIX1/4/6, MNAT1	61,151,425	T/G	1.7%	0.827	−0.49	0.09	6.31e-8	0.9%
eCO	rs12914385	15	CHRNA3/5	78,898,723	C/T	43%	1.000	0.12	0.02	2.38e-8	1.2%
CPD	rs1412076	9	NTNG2/ SETX	135,032,890	A/G	37.6%	0.999	0.10	0.02	2.24e-7	0.8%
CPD	rs9599114	13	PCDH9	66,987,131	T/C	41.7%	0.957	0.11	0.02	7.94e-8	0.8%
Cessation	rs212420	7	ATXN7L1/ CDHR3	105,496,412	C/G	6.7%	0.992	−0.60	0.11	1.50e-7	n/a
Cessation	rs207675	10	KCNMA1	79,154,537	T/C	33.6%	0..988	−0.35	0.06	5.95e-8	n/a

*UGT2B10* UDP glucuronosyltransferase family 2 member B10, *CHRNA3* cholinergic receptor nicotinic alpha 3 subunit, *CHRNA5* cholinergic receptor nicotinic alpha 5 subunit, *CEP70* centrosomal protein 70, *SIX1* SIX homeobox 1, *MNAT1* MNAT1, CDK activating kinase assembly factor, *NTNG2* Nitrin G2, *SETX* senataxin, *PCDH9* protocadherin 9, *ATXN7L1* ataxin 7 like 1, *CDHR3* cadherin related family member 3, *KCNMA1* potassium calcium-activated channel subfamily M alpha 1, * Refers to odds ratio and 95% confidence intervals (CI)

The association of the two cotinine-associated loci (UGT2B10 and CHRNA3/5) with cotinine levels at subsequent years showed that the strength of the association decays during subsequent follow up visits i.e. the *P* values increase yet they maintain the same direction of effect (Additional file 1: Table S1). This decrease in significance is directly proportional to the the decrease in sample sizes available for the analysis at follow up visits with a missing rate ranging from 8% in year 1 to 51% missing subjects at year 5 (Additional file 1: Table S2).

### Genome-wide association results for exhaled carbon monoxide (eCO) levels

The GWAS of eCO levels at year 1 included 2706 smokers (Fig. 2 and Additional file 1: Figure S5). The genomic inflation factor (λ) was 1.00. Only one region; the 15q25.1 region met genome wide significance with one SNP; intronic SNP (rs12914385) mapping to the CHRNA3 gene (*P* = 2.38 × 10^-8^) (Fig. 3 region plots). Two regions approached genome wide significance for eCO. The first was the 3q22.3 region with intronic SNP rs546764 (*P* = 7.76 × 10^-8^) mapping to the CEP70 gene. The second region was the 14q23.1 region with an intergenic SNP rs140706189 (*P* = 6.31 × 10^-8^) near the SIX1/4/6 and MNAT1 genes (Table 2).

### Genome-wide association results for cigarettes per day (CPD)

The GWAS for CPD included a total of 2854 smokers at year 1 (Fig. 2 and Additional file 1: Figure S5). The genomic inflation factor (λ) was 1.00. No regions met genome-wide significance. Two regions, however, approached this level of significance. These included the 13q21.32 region with intronic SNP rs9599114 mapping to PCDH9 gene (*P* = 7.94 × 10^-8^), and the 9q34.13 region with intergenic SNP rs1412076 (*P* = 2.24 × 10^-8^) near NTNG2 (Table 2).

### Genome-wide association results for smoking cessation

At year 5 of LHS, there were 717 sustained quitters and 2175 continuous smokers (quitters vs. smokers case control GWAS). No loci met genome-wide significance but two loci very closely approached this level of significance (Figs. 2 and 3). The strongest association was intronic SNP rs207675, which mapped to the KCNMA1 gene on 10q22.3 (*P* = 5.95 × 10^-8^). The second loci included the intronic SNP rs212420 (*P* = 1.50 × 10^-8^) near the ATXN7L1 gene on 7q22.3.

### Evaluation of previously associated SNPs for smoking behaviours

Previous reports have identified genetic loci (CHRNB3/A6 region on chromosome 8 and the CYP2A6 region on chromosome 19) that were significantly associated with CPD [33] . In the present study, both of these SNPs showed no significant association with CPD (*P* > 0.05). Additionally, we evaluated 15 SNPs that were nominally related to smoking cessation in two previous publications [28] and a number of other SNPs that were nominally associated with smoking cessation among COPD subjects [29].

Additional file 1: Table S3 shows the results of the look-up. One SNP; rs4362358 near the CHRNA3/5 genes that was related to cessation in the TAG consortium [28] was associated with eCO (*P* = 0.03) and cotinine (*P* = 0.002) in our study. Another cessation SNP in the TAG consortium; rs17178639 in SLC25A21 gene was associated with eCO (*P* = 0.007) in the present study. The two SNPs were associated with reduced cessation in the TAG consortium and were also associated with higher eCO and cotinine levels in our study. Of the cessation SNPs in COPD patients from the study of Siedlinski et al. [29], two near the IPMKP1 gene were nominally associated with cessation in our study: rs9506942, and rs9552733 with *P* = 0.005 and *P* = 0.004, respectively and with the same direction of effect.

We tested SNPs identified in our study for associations with smoking phenotypes in the TAG consortium (CPD, cessation, age of onset and ever vs. never phenotypes). Only CHRNA3/5 SNPs (for eCO and cotinine) were significant in the TAG data for both CPD and for cessation (Additional file 1: Table S4).

### Associations with lung function

We tested the SNPs identified in this study for associations with extremes of lung function: high vs. low FEV1 in heavy smokers and separately for never smokers in a large study from the UK Biobank [34]. Only the CHRNA3/5 region variants showed associations with lung function and only in the heavy smokers group (not in the never smokers). The results for lung function are shown in Additional file 1: Table S5.

## Discussion

Smoking places a huge burden on individuals and health care systems. Smoking behaviours, and consequently the risk of smoking related illnesses are at least partially genetically determined [35, 36]. The majority of previous studies on the genetics of smoking behaviour have relied on self-report, which is affected by recall bias and more importantly under-reporting bias, which may lead to an inaccurate assessment of smoking exposure. Indeed, several groups have shown that in approximately 25 to 50% of self-reported quitters, objective assays could not validate the reported smoking status [37] . It is thus crucial to accurately phenotype smoking status to properly understand the molecular mechanisms underlying nicotine addiction, metabolism, and smoking cessation.

In the current study of COPD subjects, we performed GWAS for the phenotypes of self-reported cigarettes per day (CPD) and two biochemical biomarkers of smoking: eCO and salivary cotinine. Additionally, we evaluated genetic determinants of biochemically validated smoking cessation over 5 years. Using this approach, we identified genome-wide significant loci associated with salivary cotinine on 15q.25.1 (CHRNA3/5 genes) and 4q13.2 (UTG2B10) gene). For eCO, only the 15q.25.1 locus reached genome-wide significance. The smoking cessation GWAS revealed a borderline signal in the KCNMA1 gene on 10q22.3. Finally, of all the loci identified in the current study, only the CHRNA3/5 locus showed a significant association with lung function in heavy smokers (but not in never smokers) from the general population.

Genetic determinants of smoking can be related to smoking intensity (addiction), metabolism or both. The metabolism of nicotine involves several enzymatic pathways. Approximately 10% of nicotine is excreted unchanged in the urine. The majority of nicotine (~ 80%) is converted to cotinine in two steps: initial metabolism, which is mediated by the cytochrome P450, family 2, subfamily A, poly-peptide 6 (CYP2A6) enzyme, followed by conjugation by aldehyde oxidase [38]. After these two steps cotinine is further metabolised by CYP2A6 to 3-hydroxycotinine. Oxidation and glucuronidation processes account for the remaining 10% of the metabolic process [38]. We found significant association of salivary cotinine with SNPs in UDP glucuronosyltransferase family 2 member B10 (UGT2B10), which catalyses both nicotine and cotinine glucuronidation in smokers.

Previous GWAS for cotinine in urine, plasma or serum have all identified the UGT2B10 region [16, 39] with stronger associations reported for urinary cotinine levels. In the current study, we replicated the association signal for SNPs in the UGT2B10 region for association with salivary cotinine. Smokers are thought to self-titrate their nicotine to meet their physiological need [40] (i.e. high metabolizers are likely to smoke more). If this hypothesis were true, then we would expect variations in the rates of metabolism (and hence consumption) to be associated with smoking related diseases/phenotypes. However, we failed to find any significant associations between SNPs in the UGT2B10 gene region with impaired lung function in smokers. We may not have sufficient power to detect a subtle effects of this locus; alternatively the two mechanisms (metabolism and consumption) may not be directly linked as previously suggested [34]. The association results of the cotinine and eCO-associated SNPs with CPD in the same individuals are shown in Additional file 1: Table S6. The results indeed show that the UGT2B10 variant is not associated with CPD (*P* = 0.28), arguing against the notion that variation in metabolism of nicotine may affects smoking behaviour.

Perhaps the most widely studied and reported region for smoking is CHRNA3/5 on 15q25. The associations of this region in the current study are with biochemical biomarkers of smoking (eCO and cotinine). In the present study, the CHRNA3/5 variants were associated with salivary cotinine as well as eCO levels. Importantly, these variants were also significantly related to CPD (*P* < 0.05), suggesting this genetic region modulates cigarette consumption (Additional file 1: Table S6). However, this locus was not associated with smoking cessation in LHS participants, suggesting that other factors are involved in quitting. Other previously reported loci in CYP2A6 and CHRNB3/CHRNA6 genes could not be replicated in our study. However, as previously noted [35, 36], the strength of the relationship between these loci and smoking is relatively modest and may require much larger sample sizes to be detected.

We identified a suggestive signal (*P* = 5.95 × 10^-8^) for smoking cessation in the potassium calcium-activated channel subfamily M alpha 1 (KCNMA1) gene which is important for the control of smooth muscle tone and neuronal excitability [41, 42]. The association between the KCNMA1 variant with cessation in our study could not be replicated in the cessation GWAS from the TAG consortium [28]. This could be due to the fact that our study used a biochemically verified smoking status; whereas the previous studied relied only on self-report. Our data are in keeping with a previously published study. A GWAS in Australian and Dutch populations identified SNPs in the KCNMA1 gene, which were significantly associated with nicotine dependence (rs592676, *p* = 8.91 × 10^-6^). In our study, the same SNP was strongly associated with cessation (*P* = 5.6 × 10^-7^). Interestingly, a drug repositioning study that integrated disease and drug expression profiles identified KCNMA1 as a potential molecular target for lobeline: a natural alkaloid that has been used as a smoking cessation aid [43] as well as for amphetamine and cocaine addictions [44] KCNMA1 is a target for the FDA approved drug, chlorzoxazone, which is a centrally acting muscle relaxant. Chlorzoxazone acts as an activator of a calcium-activated potassium channel [45] and is commonly used as a probe drug to phenotype CYP2E1 activity and its metabolism is strongly accelerated by cigarette smoking [46]. Another study proposed chlorzoxazone as a potential treatment for alcohol addiction [47]. At the gene expression level, NHBE cells exposed to nicotine-containing e-cigarette vapour demonstrate decreased expression of KCNMA1 [48], while in human lung tissue smokers have significantly higher expression compared to never smokers (1.5 fold change, *P* = 3.11 × 10^− 08^) [49]. Finally, a genome-wide study identified differential hydroxymethylation of potassium channel genes, including KCNMA1, in the nucleus accumbens in methamphetamine addiction and abstinence [50]. Taken together, these data suggest the KCNMA1 association with smoking cessation is biologically plausible with the potential for drug repurposing.

This study has several limitations. The sample size may have been too small to detect novel loci for smoking biomarkers or cessation. On the other hand, the use of precise biochemical phenotypes on the other hand likely improved the specificity of the smoking cessation signal. Furthermore, and in line with most published GWASs, the proportion of variance explained by the identified variants is small.

## Conclusion

In conclusion, we identified genetic loci associated with eCO and cotinine in COPD patients. Our study strongly support the need to use objective measures of smoking to capture the genetic contribution to smoking in these studies. The KCNMA1 region association with smoking cessation represents a potential target for drug discovery and repurposing which warrants further studies.

## Additional file

Additional file 1: Supplementary Figures and Tables. (DOCX 1183 kb)
