# Supplementary material for: The genetics of smoking in individuals with chronic obstructive pulmonary disease
Source: Respir Res. 2018 Apr 10;19:59. doi: 10.1186/s12931-018-0762-7 (PMC5892035; doi:10.1186/s12931-018-0762-7)
Supplement: Supplementary file 1 — Supplementary Figures and Tables. (DOCX 1183 kb) [file 12931_2018_762_MOESM1_ESM.docx]

**The Genetics of Smoking in Individuals with Chronic Obstructive Pulmonary Disease**

Ma’en Obeidat^1^, Guohai Zhou^1^, Xuan Li^1^, Nadia N. Hansel^2^, Nicholas Rafaels^3^, Rasika Mathias^4^, Ingo Ruczinski^5^, Terri H. Beaty^6^, Kathleen C. Barnes^3^, Peter D. Paré^1,7^, and Don D. Sin^1,7^

**Supplementary Materials**

Contents

[Supplementary figures 2](#_Toc509839561)

[Supplementary Figure 1. Phenotype distribution before and after Z score transformation 2](#_Toc509839562)

[Supplementary Figure 2. The correlation between eCO, cotinine and CPD among smokers at year 1 of LHS. 3](#_Toc509839563)

[Supplementary Figure 3: Cotinine levels vs. FEV1 in litres at each annual visit. 4](#_Toc509839564)

[Supplementary Figure 2: Cotinine levels at year 5 and FEV1 decline between years 1-5 5](#_Toc509839565)

[Supplementary Figure 5: QQ plots for smoking behaviour GWAS 6](#_Toc509839566)

[Supplementary Tables 7](#_Toc509839567)

[Supplementary Table 1: The association of the cotinine associated SNPs with cotinine levels at baseline and years 1-5 of LHS. 7](#_Toc509839568)

[Supplementary Table 2: The sample sizes and the missing rate for cotinine measurements at baseline and years 1-5 of LHS. 8](#_Toc509839569)

[Supplementary Table 3: Evaluation of previously published SNPs for smoking behaviour in the current study. 10](#_Toc509839570)

[Supplementary Table 4: Evaluation of SNPs identified in this study for association with publically available GWAS data from the TAG consortium. 11](#_Toc509839571)

[Supplementary Table 5: Look-up of smoking behaviour loci in the UK BilEVE study for extremes of lung function in heavy and never smokers separately. 12](#_Toc509839572)

[Supplementary Table 6: The association of eCO and cotinine GWAS hits in LHS with cigarettes per day (CPD). 13](#_Toc509839573)

[References 13](#_Toc509839574)

# Supplementary figures

**
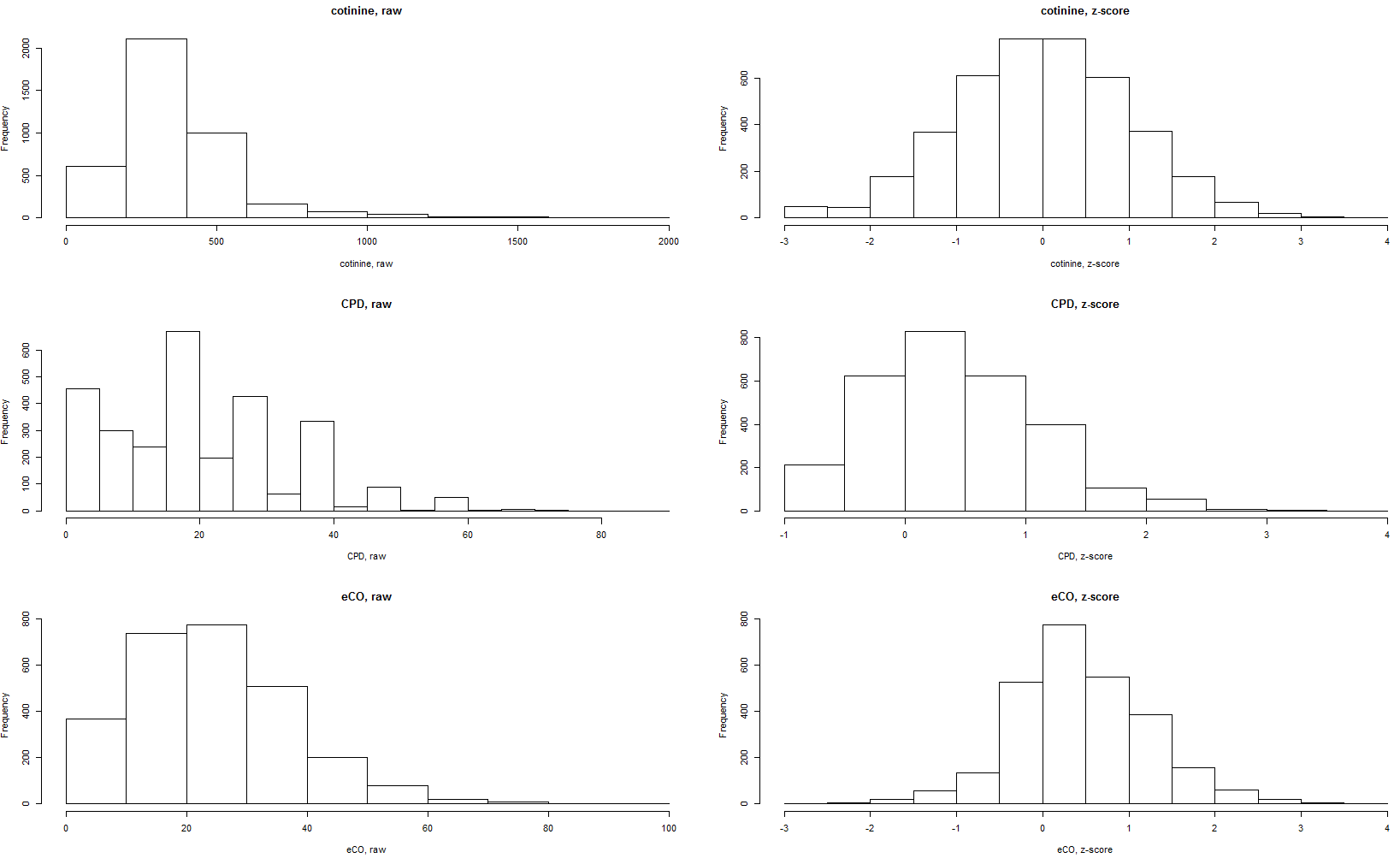
**

## Figure S1. Phenotype distribution before and after Z score transformation


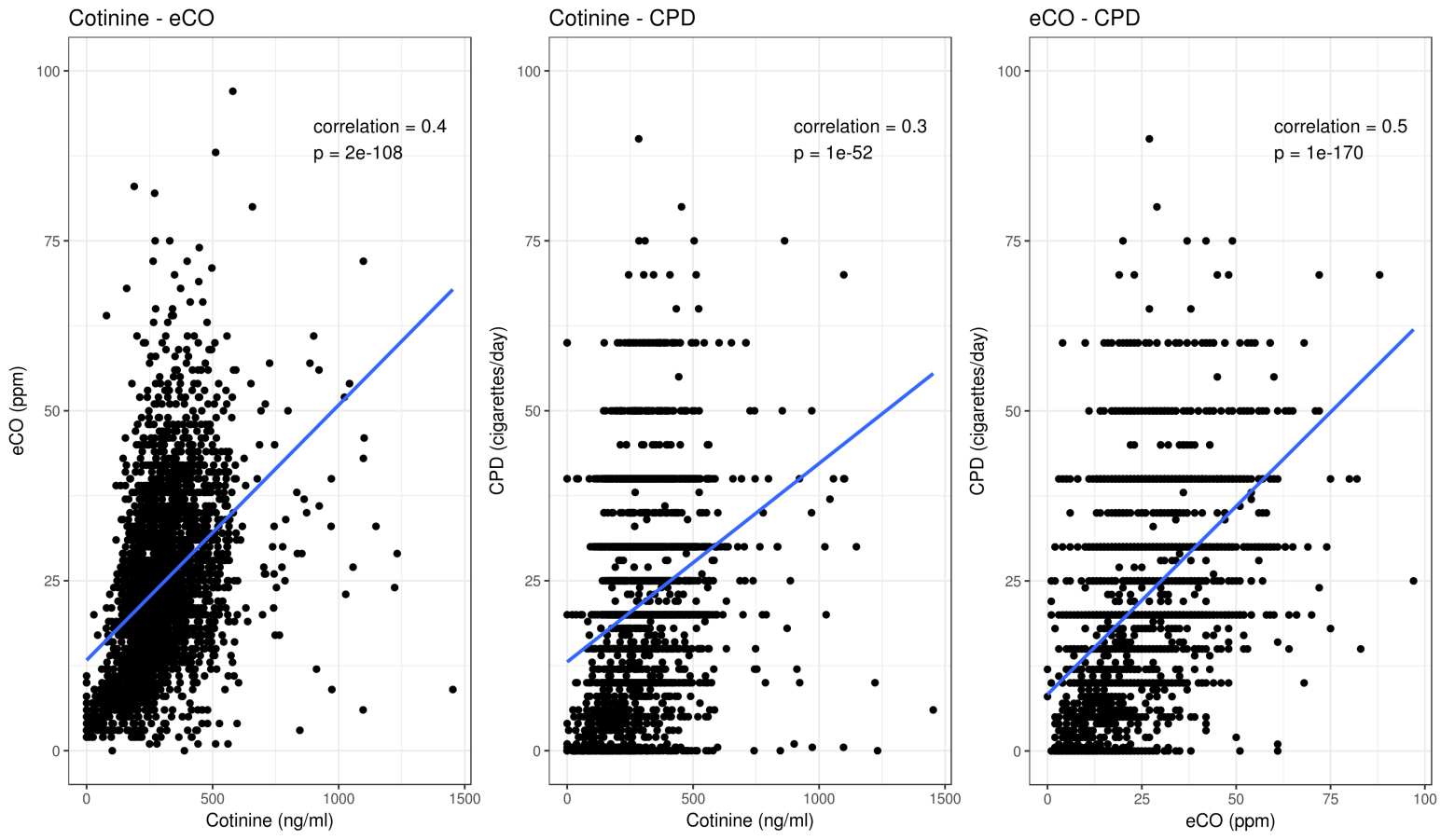


## Figure S2. The correlation between eCO, cotinine and CPD among smokers at year 1 of LHS.

The correlation and p values are using Pearson's correlation.


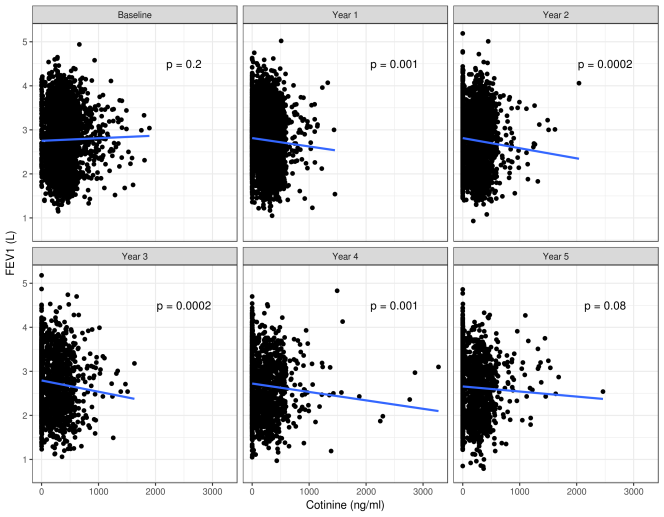


## Figure S3: Cotinine levels vs. FEV1 in litres at each annual visit.


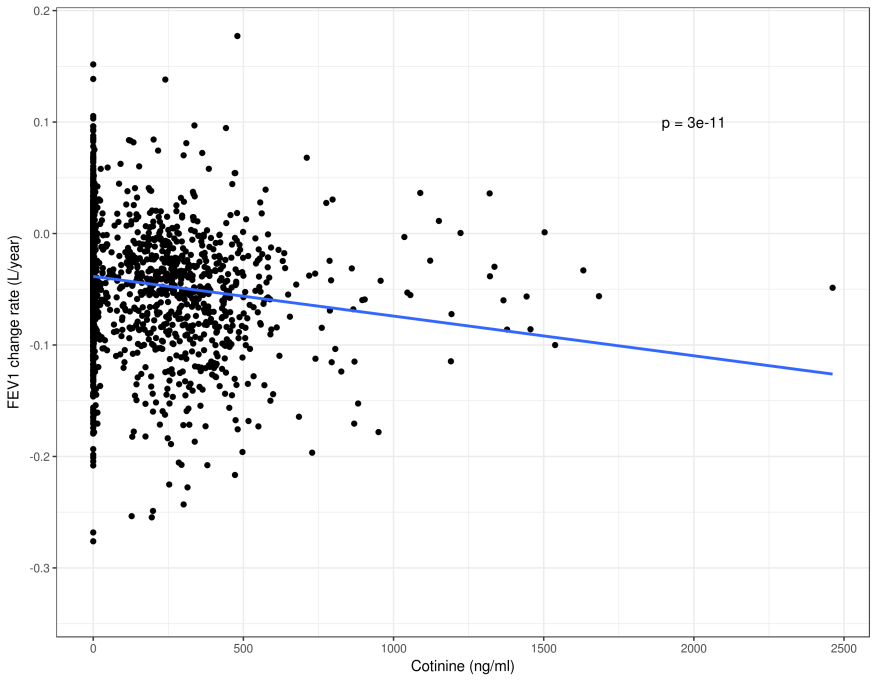


## Figure S4: Cotinine levels at year 5 and FEV1 decline between years 1-5

## Figure S5: QQ plots for smoking behaviour GWAS

# Supplementary Tables

| **SNP** | **gene** | **visit** | **estimate** | **std.error** | **p.value** |
| --- | --- | --- | --- | --- | --- |
| rs10023464 | UGT2B10 | Baseline | 0.243668 | 0.037049 | 5.42E-11 |
| rs10023464 | UGT2B10 | Year 1 | 0.092677 | 0.037269 | 0.012936 |
| rs10023464 | UGT2B10 | Year 2 | 0.093927 | 0.042759 | 0.028132 |
| rs10023464 | UGT2B10 | Year 3 | -0.02014 | 0.048306 | 0.676857 |
| rs10023464 | UGT2B10 | Year 4 | 0.017064 | 0.045884 | 0.710019 |
| rs10023464 | UGT2B10 | Year 5 | 0.016671 | 0.043923 | 0.704327 |
| rs9788721 | CHRNA3/5 | Baseline | -0.11853 | 0.02274 | 1.96E-07 |
| rs9788721 | CHRNA3/5 | Year 1 | -0.07285 | 0.022662 | 0.001317 |
| rs9788721 | CHRNA3/5 | Year 2 | -0.09838 | 0.0265 | 0.000209 |
| rs9788721 | CHRNA3/5 | Year 3 | -0.05501 | 0.029837 | 0.065409 |
| rs9788721 | CHRNA3/5 | Year 4 | -0.02116 | 0.029498 | 0.473255 |
| rs9788721 | CHRNA3/5 | Year 5 | -0.04246 | 0.027853 | 0.127542 |

## Table S1: The association of the cotinine associated SNPs with cotinine levels at baseline and years 1-5 of LHS.

| **visit** | **Sample size** | **Missing rate** |
| --- | --- | --- |
| Baseline | 4024 | 1.90% |
| Year 1 | 3759 | 8.36% |
| Year 2 | 2637 | 35.71% |
| Year 3 | 1832 | 55.34% |
| Year 4 | 1840 | 55.14% |
| Year 5 | 2000 | 51.24% |

## Table S2: The sample sizes and the missing rate for cotinine measurements at baseline and years 1-5 of LHS.

| SNP | Published reference | Gene | Published phenotype | ALT | REF | beta_CPD | P_CPD | beta_eCO | P_eCO | beta_cot | P_cot | beta_sustQuit  _vs_smoker | P_sustQuit  _vs_smoker |
| --- | --- | --- | --- | --- | --- | --- | --- | --- | --- | --- | --- | --- | --- |
| rs6474412 | Thorgeirsson et al. [[1](#_ENREF_1)] | CHRNB3/ CHRNA6 | CPD | T | C | 0.039734 | 0.09936 | 0.033271 | 0.198851 | 0.025418 | 0.344524 | -0.08436 | 0.257972 |
| rs1329650 | TAG [[2](,#_ENREF_2)] | HECTD2-AS1 | CPD | T | G | 0.028346 | 0.205673 | -0.03109 | 0.198935 | 0.020757 | 0.402262 | 0.048032 | 0.483147 |
| rs1028936 | TAG[[2](,#_ENREF_2)] | HECTD2-AS1 | CPD | C | A | 0.021331 | 0.42843 | -0.02257 | 0.438123 | 0.057395 | 0.052864 | 0.020708 | 0.803026 |
| rs215614 | Thorgeirsson et al. [[1](#_ENREF_1)] | PDE1C | CPD | A | G | -0.00771 | 0.706808 | -0.00188 | 0.932318 | 0.035554 | 0.121676 | -0.00773 | 0.903395 |
| rs3733829 | TAG[[2](,#_ENREF_2)] | EGLN2/CYP2A6 | CPD | G | A | 0.008667 | 0.676246 | 0.031885 | 0.155308 | 0.036414 | 0.11866 | -0.02409 | 0.713605 |
| rs3025343 | TAG[[2](,#_ENREF_2)] | DBH | Cessation | A | G | 0.016038 | 0.599828 | 0.008061 | 0.808107 | 0.021464 | 0.52708 | -0.04275 | 0.651118 |
| rs1782352 |  |  | Cessation | T | C | 0.048776 | 0.30244 | 0.070724 | 0.167295 | 0.085279 | 0.114751 | -0.05962 | 0.69374 |
| rs698782 |  |  | Cessation | C | T | -0.03689 | 0.320362 | -0.01735 | 0.663817 | -0.04694 | 0.260618 | -0.1098 | 0.34718 |
| rs10205020 |  |  | Cessation | G | A | 0.023147 | 0.356499 | 0.020568 | 0.446283 | -0.03636 | 0.196057 | 0.003311 | 0.966149 |
| rs6570644 |  |  | Cessation | G | A | -0.02441 | 0.278387 | -0.02907 | 0.231014 | -0.04327 | 0.084322 | -0.02229 | 0.751853 |
| rs10234489 |  |  | Cessation | C | T | 0.004842 | 0.843483 | -0.02229 | 0.400018 | 0.016155 | 0.555379 | 0.048762 | 0.518767 |
| rs17250591 |  |  | Cessation | A | G | -0.01591 | 0.435729 | 0.028434 | 0.195404 | -0.00338 | 0.882327 | -0.04492 | 0.479859 |
| rs6471277 |  |  | Cessation | C | T | 0.01153 | 0.578153 | -0.02195 | 0.324735 | -0.03794 | 0.101159 | 0.03033 | 0.636784 |
| rs3025360 |  |  | Cessation | A | G | 0.016102 | 0.598445 | 0.007914 | 0.811601 | 0.021856 | 0.519553 | -0.04277 | 0.651096 |
| rs2582895 |  |  | Cessation | A | C | 0.012693 | 0.535438 | 0.019813 | 0.369256 | -0.00719 | 0.754577 | -0.08012 | 0.208831 |
| rs10895192 |  |  | Cessation | G | T | -0.01137 | 0.57435 | 0.002177 | 0.920677 | 0.021373 | 0.34509 | 0.011823 | 0.851316 |
| rs17178639 |  |  | Cessation | G | A | -0.07673 | 0.151462 | -0.15701 | **0.007335** | -0.01178 | 0.84457 | 0.040559 | 0.810594 |
| rs1797227 |  |  | Cessation | G | A | -0.01848 | 0.357836 | -0.03289 | 0.13018 | 0.003829 | 0.865251 | 0.09091 | 0.147655 |
| rs4362358 |  |  | Cessation | T | C | 0.034611 | 0.102128 | 0.049647 | **0.030041** | 0.072712 | **0.002139** | 0.028307 | 0.665427 |
| rs6134154 |  |  | Cessation | G | A | 0.041403 | 0.254092 | 0.055581 | 0.158047 | -0.06314 | 0.118206 | -0.18191 | 0.118086 |
| rs10794613 | Siedlinski et al. [[3](#_ENREF_3)] |  | Cessation in COPD subjects | G | C | 0.04347 | 0.075458 | 0.045781 | 0.083682 | 0.014351 | 0.60215 | -0.09439 | 0.213521 |
| rs1896376 |  |  | Cessation in COPD subjects | T | C | -0.04603 | 0.293362 | -0.05449 | 0.245759 | -0.04214 | 0.397255 | -0.15843 | 0.269318 |
| rs727417 |  |  | Cessation in COPD subjects | C | G | 0.040036 | 0.35399 | 0.038813 | 0.404505 | 0.019243 | 0.696239 | 0.229115 | 0.110587 |
| rs10861185 |  |  | Cessation in COPD subjects | C | A | 0.00689 | 0.732991 | -0.01513 | 0.487531 | -0.02521 | 0.259655 | 0.020645 | 0.739917 |
| rs9506942 |  |  | Cessation in COPD subjects | G | C | 0.014256 | 0.477942 | 0.011443 | 0.597674 | -0.01921 | 0.396107 | -0.17621 | **0.005477** |
| rs9552733 |  |  | Cessation in COPD subjects | A | G | 0.012374 | 0.53862 | 0.007248 | 0.738751 | -0.01491 | 0.511311 | -0.18166 | **0.004387** |
| rs13064954 |  |  | Cessation in COPD subjects | A | G | 0.024058 | 0.599023 | 0.002048 | 0.96682 | -0.09742 | 0.062573 | 0.020352 | 0.887752 |
| rs9866141 |  |  | Cessation in COPD subjects | T | C | 0.009146 | 0.842755 | -0.00692 | 0.889053 | -0.09702 | 0.064072 | 0.043191 | 0.76273 |
| rs1165640 |  |  | Cessation in COPD subjects | T | C | -0.05948 | 0.085629 | 0.004085 | 0.91328 | -0.08317 | **0.037072** | 0.119357 | 0.291841 |

## Table S3: Evaluation of previously published SNPs for smoking behaviour in the current study.

| **Phenotype** | **SNP** | **Proxy SNP**  **(LD R^2)** | **Chr** | **Gene(s)** | **LHS Beta (P)** | **Beta (P) CPD in TAG** | **Beta (P) Cessation in TAG** | **Beta (P) Age Onset in TAG** | **Beta (P) ever vs. never in TAG** |
| --- | --- | --- | --- | --- | --- | --- | --- | --- | --- |
| Cotinine | rs10023464 | - | 4 | UGT2B10 | 0.25 (1.27e-11) | 0.24 (9.16e-02) | -0.02 (3.86e-01) | -0.01 (**4.09e-02**) | -0.02 (2.54e-01) |
| Cotinine | rs9788721 | - | 15 | CHRNA3/5 | -0.12 (3.49e-07) | -0.99 (**4.14e-31**) | 0.07 (**3.51e-05**) | 0.00 (4.24e-01) | 0.00 (9.83e-01) |
| eCO | rs546764 | rs17484792 (0.51) | 3 | CEP70 | 0.39 (7.76e-08) | -0.97 (3.79e-01) | -0.05 (8.73e-01) | 0.01 (6.38e-01) | 0.03 (8.17e-01) |
| eCO | rs140706189 | rs4151415 (0.88) | 14 | SIX1/4/6, MNAT1 | -0.49 (6.31e-08) | 0.22 (5.84e-01) | 0.09 (2.60e-01) | -0.01 (1.38e-01) | 0.02 (7.62e-01) |
| eCO | rs12914385 |  | 15 | CHRNA3/5 | 0.12 (2.38e-08) | 1.02 (4**.23e-35**) | -0.08 (**1.82e-06**) | -0.00 (3.68e-01) | 0.00 (7.96e-01) |
| CPD | rs1412076 |  | 9 | NTNG2/ SETX | 0.10 (2.24e-07) | -0.11 (1.71e-01) | 0.01 (5.54e-01) | 0.00 (8.99e-01) | 0.01 (2.86e-01) |
| CPD | rs9599114 | rs9317587 (0.94) | 13 | PCDH9 | 0.11 (7.94e-08) | -0.02 (8.34e-01) | -0.00 (8.21e-01) | 0.00 (8.27e-01) | -0.01 (2.49e-01) |
| Cessation | rs212420 | rs212414 (0.93) | 7 | ATXN7L1/ CDHR3 | -0.60 (1.50e-07) | 0.03 (8.62e-01) | 0.03 (3.10e-01) | 0.00 (8.04e-01) | -0.02 (2.71e-01) |
| Cessation | rs207675 |  | 10 | KCNMA1 | -0.36 (5.95e-08) | -0.09 (3.23e-01) | -0.00 (8.89e-01) | 0.00 (8.70e-01) | 0.02 (6.71e-02) |

## Table S4: Evaluation of SNPs identified in this study for association with publically available GWAS data from the TAG consortium.

| Phenotype | SNP | Proxy SNP  (LD R^2) | Chr | Gene(s) | Position (hg19) | Beta (P)  LHS GWAS | Beta (P) UKBiLEVE_ Low_FEV1_vs_high_FEV1_heavy _smokers | Beta (P) UKBiLEVE_ Low_FEV1_vs_high_FEV1_never _smokers |
| --- | --- | --- | --- | --- | --- | --- | --- | --- |
| Cotinine | rs10023464 | - | 4 | UGT2B10 | 69659738 | 0.25 (1.27e-11) | 0.03 (4.94e-01) | -0.00 (9.53e-01) |
| Cotinine | rs9788721 | - | 15 | CHRNA3/5 | 78802869 | -0.12 (3.49e-07) | -0.17 (**2.52e-10**) | -0.02 (4.42e-01) |
| eCO | rs546764 | rs17484792 (0.51) | 3 | CEP70 | 138294336 | 0.39 (7.76e-08) | -0.05 (4.66e-01) | -0.11 (1.05e-01) |
| eCO | rs140706189 | rs4151415 (0.88) | 14 | SIX1/4/6, MNAT1 | 61151425 | -0.49 (6.31e-08) | 0.02 (8.36e-01) | -0.04 (7.01e-01) |
| eCO | rs12914385 |  | 15 | CHRNA3/5 | 78898723 | 0.12 (2.38e-08) | 0.17 (**2.73e-11**) | 0.01 (6.68e-01) |
| CPD | rs1412076 |  | 9 | NTNG2/ SETX | 135032890 | 0.10 (2.24e-07) | 0.01 (5.77e-01) | 0.02 (5.38e-01) |
| CPD | rs9599114 | rs9317587 (0.94) | 13 | PCDH9 | 66987131 | 0.11 (7.94e-08) | 0.00 (8.94e-01) | 0.02 (4.15e-01) |
| Cessation | rs212420 | rs212414 (0.93) | 7 | ATXN7L1/ CDHR3 | 105496412 | -0.36 (5.95e-08) | 0.02 (7.64e-01) | 0.00 (9.67e-01) |
| Cessation | rs207675 |  | 10 | KCNMA1 | 79154537 | -0.60 (1.50e-07) | 0.00 (9.53e-01) | 0.00 (9.14e-01) |

## Table S5: Look-up of smoking behaviour loci in the UK BilEVE study for extremes of lung function in heavy and never smokers separately.

| **Phenotype** | **SNP** | **Beta** | **SE** | **P** |
| --- | --- | --- | --- | --- |
| Cotinine | rs10023464 | -0.036 | 0.033 | 2.83E-01 |
| Cotinine | **rs9788721** | -0.046 | 0.020 | **2.28E-02** |
| eCO | **rs546764** | 0.258 | 0.067 | **1.08E-04** |
| eCO | **rs140706189** | -0.204 | 0.085 | **1.73E-02** |
| eCO | **rs12914385** | 0.055 | 0.020 | **6.47E-03** |
| Cessation | rs212420 | -0.003 | 0.042 | 9.35E-01 |
| Cessation | rs207675 | -0.017 | 0.022 | 4.21E-01 |

## Table S6: The association of eCO and cotinine GWAS hits in LHS with cigarettes per day (CPD).

SNPs in bold are associated with CPD at P<0.05.

# References

1. Thorgeirsson TE, Gudbjartsson DF, Surakka I, Vink JM, Amin N, Geller F: **Sequence variants at CHRNB3-CHRNA6 and CYP2A6 affect smoking behavior.** *Nat Genet* 2010, **42:**448 - 453.

2. Consortium" TaG: **Genome-wide meta-analyses identify multiple loci associated with smoking behavior.** *Nat Genet* 2010, **42:**441-447.

3. Siedlinski M, Cho MH, Bakke P, Gulsvik A, Lomas DA, Anderson W, Kong X, Rennard SI, Beaty TH, Hokanson JE, et al: **Genome-wide association study of smoking behaviours in patients with COPD.** *Thorax* 2011, **66:**894-902.
